# “I know it will make me feel better”: a grounded theory of sea gazing and well-being for women in midlife

**DOI:** 10.1080/17482631.2025.2527883

**Published:** 2025-07-07

**Authors:** Sarah L. Hurdman, Donna C. Jessop, Megan Hurst, Tom L. Farsides

**Affiliations:** School of Psychology, University of Sussex, Brighton, UK

**Keywords:** Sea, blue spaces, well-being, midlife women, grounded theory

## Abstract

**Background:**

Midlife has been identified as a period of diminished health and well-being among women. A growing body of evidence has documented positive links between interactions with the sea and well-being. To date, most qualitative researchers have focused on the experience and benefits of activities taking place in, or on, the water. Hence, this study aimed to explore the experiences of looking out to sea from the land (“sea gazing”), and how it may be related to well-being, for women in midlife.

**Methods:**

Grounded theory methodology was used to develop a substantive theory of the relationship between sea gazing and well-being. Data were collected from 15 coastal-dwelling women, aged between 45–64 years old.

**Results:**

The proposed theory explains a self-reinforcing, cyclical process in which sea gazing is used as a habitual self-care practice within overall well-being management. The theory was constructed around a core category of *habitually reconnecting with the sea to help manage well-being*.

**Conclusions:**

Sea gazing from the land may offer a simple, affordable and accessible way for midlife women to gain well-being benefits from the sea . These results may be of interest to policymakers and healthcare professionals concerned with facilitating positive well-being outcomes for women in midlife.

For women, middle age has been characterized as a period of challenge, transition and decline. Nevertheless, adopting positive health behaviours in midlife has been shown to help mitigate the mental and physiological risk factors identified in women at this life stage (Avis et al., 2021). However, there remains a paucity of well-being research focusing on women in midlife (Kopanidis & Reid, 2024). Spending time in nature is one activity shown to promote well-being outcomes in the general population, the old and the young (Banwell et al., 2024; Burke et al., 2023). Visiting the coast, and living in close proximity to the sea, have been associated with positive mental and physical well-being outcomes (White et al., 2020). However, the impact of different types of engagement with the sea among different demographic groups is not well understood. Qualitative researchers have begun to study the well-being impact of activities taking place in, or on, the sea (e.g., Denton & Aranda, 2020; Lisahunter & Stoodley, 2021; Pipere et al., 2020). However, engaging with the sea from the land, such as when looking out to sea (referred to as *sea gazing*), has yet to be fully explored. Hence, this study used grounded theory methodology (Corbin & Strauss, 2015) to develop an explanatory theory of the relationship between sea gazing from the land and well-being for women in midlife. Given evidence that midlife is a critical window for optimizing healthy functioning for women in later life (Avis et al., 2021), understanding if, and how, sea gazing may benefit well-being for this age group could have significant public health benefits.

## Women in midlife

Women in midlife often experience adverse changes across numerous physical and psychological health domains (El Khoudary et al., 2019). Although the literature defines this age group flexibly, it typically includes the years 45–64. This life stage has been associated with poorer physiological and physical functioning, the onset of chronic disease, a higher incidence of depression and anxiety and a range of adverse menopause-related symptoms including vasomotor problems, sleep complaints and cognitive difficulties (Bondarev et al., 2021; El Khoudary et al., 2019; Infurna et al., 2020). For many women, personal, family and societal stressors may be magnified during midlife. Such stressors may include balancing ageing parent and child caregiving, changes in relationship status and financial and work pressures (Infurna et al., 2020; Lachman, 2004; Thomas et al., 2018). Nevertheless, many health risk factors are modifiable in middle age, and their effects can be mitigated by engaging in positive health and well-being behaviours (Avis et al., 2021; Lafortune et al., 2016). However, whilst optimizing physical and psychological well-being in middle age has been shown to be a predictor of healthy functioning in later decades (Avis et al., 2021; Harlow & Derby, 2015), women’s midlife remains a significantly under-researched life stage (Infurna et al., 2020; Kopanidis & Reid, 2024).

## Conceptualising well-being

The conceptualization and measurement of well-being has been the subject of much debate in the literature, with the terms *health* and *well-being* argued to overlap (Lomas, 2015; Trigwell et al., 2014). Perspectives on well-being typically draw a distinction between hedonic and eudaimonic conceptions of what constitutes a life well lived (Huta & Waterman, 2014; Ryff et al., 2021). Hedonic frameworks focus on pleasure, enjoyment and the absence of discomfort; whereas eudaimonic approaches centre on what is worth pursuing in life (e.g., personal growth, meaning in life, authenticity, and excellence). However, the experience and conceptualization of well-being is argued to seldom be the same across individuals and can change over time and across contexts (Edwards et al., 2016). Hence, qualitative researchers investigating the link between nature and well-being have recommended the exploration of individual experiences, thoughts and feelings alongside established well-being frameworks (Bell et al., 2015). Thus, this study advocates the use of in-depth qualitative methods to understand how women may uniquely construe well-being and how different natural settings, such as the sea, may play a role in meeting their well-being needs.

## Blue spaces and well-being

Spending time in natural environments is one factor shown to positively impact health and well-being outcomes across the general population (Banwell et al., 2024; Burke et al., 2023; Geneshka et al., 2021), in old age (Helbich et al., 2019; Huang et al., 2022) and in children and adolescents (Liu & Green, 2023; Ye et al., 2022). To date, most research has focused on the benefits of green spaces (such as gardens, parks, and woodland), but more recently the relationship between blue spaces (bodies of coastal and inland water) and well-being has begun to be explored in the scientific literature. Visiting and living near blue spaces has been associated with better self-reported mental health, general health and subjective well-being (for reviews see Britton et al., 2020; Gascon et al., 2017, Smith et al., 2021; White et al., 2020). Indeed, empirical studies have suggested that the well-being benefits of visiting blue spaces may be greater than spending time in green nature. Experience sampling studies have reported the highest levels of subjective well-being for people at marine or coastal margins—significantly higher than for urban locations, and for those in green areas (MacKerron & Mourato, 2013; Stieger et al., 2022). Perhaps unsurprisingly, recent research suggests that *direct exposure* to blue spaces may be a more important predictor of the health and well-being benefits of outdoor water than simply living close by (Elliott et al., 2023; White et al., 2021). Making recreational visits to blue spaces more often, and for longer periods, has been more strongly associated with improved well-being and reduced mental distress than residential proximity (Garrett et al., 2019; White et al., 2021. Overall, there is growing correlational evidence that spending time in blue spaces is linked with benefits to health and well-being, and that these benefits may increase with frequency and duration.

Alongside the quantitative literature, findings from qualitative studies have begun to illuminate our understanding of *how* different blue spaces are experienced and associated with well-being outcomes. The emotional, aesthetic and spiritual qualities of specific blue spaces have been claimed to promote deep emotional attachment and a sense of belonging (e.g., Coleman & Kearns, 2015; Game & Metcalfe, 2011; Kearns & Collins, 2012; Severin et al., 2022). Visiting the coast has been found to promote a sense of connection with the natural world and bring meaning through relating to something greater and more permanent (e.g., Game & Metcalfe, 2011; Jarratt & Sharpley, 2017). Indeed, Diamond et al. (2024) found that people can develop a unique attachment to coastal areas that may not be transferred to other natural environments. They also suggested that such coastal place attachment is both a product and a facilitator of psychological, physical and social well-being. Overall, empirical qualitative studies have found coastal visits to be immersive, therapeutic and salutogenic experiences that can promote hedonic and eudaimonic well-being (e.g., Bell et al., 2015; Jarratt & Sharpley, 2017; Kelly, 2018).

## Pathways linking blue spaces and well-being

Researchers have also begun to explore the causal mechanisms that may underpin the relationship between blue spaces and well-being. Studies have typically used pathway frameworks originating in the green space literature, most commonly, restoration (psychological and physiological), social connection and physical activity (Hartig et al., 2014; Markevych et al., 2017). Overall, greater direct exposure to blue spaces has been associated with higher levels of restoration and physical activity, but the evidence is inconclusive regarding the mediating role of social interaction (Gascon et al., 2017; Georgiou et al., 2021; Nicolosi et al., 2021; Vert et al., 2020). Qualitative studies have suggested that pathways of physical activity and social interaction are involved in facilitating well-being benefits but have particularly highlighted the restorative role of spending time in blue spaces. For example, researchers have found that mindful engagement with the multisensory nature of coastal settings can clear the mind of cognitive noise, promote positive emotions and reduce stress (e.g., Bell et al., 2015; Kelly, 2018; Severin et al., 2022).

Additionally, qualitative studies have suggested that experiencing an emotional, cognitive and/or spiritual connection to specific blue spaces may serve as a causal pathway to well-being (Coleman & Kearns, 2015; Diamond et al., 2024; Game & Metcalfe, 2011; Jarratt & Sharpley, 2017; Severin et al., 2022). In support of this, quantitative researchers have demonstrated a link between connectedness to nature and both hedonic and eudaimonic well-being outcomes. However, such studies have typically focused only on the benefits of connectedness to *green* spaces (e.g., H. Liu et al., 2022) or have not specified the type of natural environment being studied (e.g., Capaldi et al., 2014; Martin et al., 2020; Pritchard et al., 2020). Yet researchers in the field have acknowledged that the relationship between *nature connectedness* (Mayer & Frantz, 2004) and well-being is idiosyncratic, and may vary in different contexts, and over time (Cleary et al., 2017; Martin et al., 2020; Pritchard et al., 2020). Indeed, qualitative evidence to date is consistent with the notion that connectedness to blue spaces, particularly the sea, may be experienced as distinct from connectedness to other natural environments (e.g., Diamond et al., 2024; Jarratt & Sharpley, 2017; Severin et al., 2022).

Overall, whilst the literature offers some insight into the potential pathways between blue space and well-being, researchers have yet to clarify how they may vary between individuals and specific types of engagement with nature (Martin et al., 2020; Richardson et al., 2021). As such, qualitative methodologies may offer the acuity required to inform our understanding of the complexities of these relationships and the causal mechanisms involved.

## Engaging with the sea

Whilst visiting outdoor water in general has been associated with health benefits, less is known about *how* the different ways in which people engage with coastal blue space may be experienced and may differentially promote well-being outcomes. Yet evidence from the green space literature suggests that the health and well-being benefits of nature are not simply linearly related to the amount of time spent within it (Martin et al., 2020). The type and quality of contact, and the extent to which individuals experience a cognitive and emotional connection with nature, have been shown to influence the strength of the association between exposure to nature and well-being (Liu et al., 2022; Martin et al., 2020; Richardson et al., 2021). Qualitative studies have begun to shed light on how different types of engagements with the sea may be experienced, and how they may relate to well-being. To date, researchers have typically focused on the experience and benefits of participating in activities requiring immersion in or on water. For example, studies have found sea swimming to be a transformative, multisensory and embodied experience that connects individuals to specific places—and the natural world in general—and promotes physical, mental and social well-being (e.g., Bates & Moles, 2021; Denton & Aranda, 2020; Foley, 2017). Surfing has been found to promote powerful feelings of freedom, amazement and happiness, and be used by participants as a coping mechanism when dealing with psychological distress (e.g., Lisahunter & Stoodley, 2021); whilst sailing has been shown to shift focus from *self* to *environment* and facilitate existential well-being (e.g., Pipere et al., 2020). However, existing literature has rarely explored the experience of engaging with the sea *from the land* - and how it may impact well-being—in any depth. In addition, participating in activities requiring immersion in or on the sea may not appeal to everyone, often requires specific skills and physical abilities and may be costly in terms of time and money (Ford & Brown, 2006; Jackson, 1983; Sailors & Weaving, 2024; Sotiriadou et al., 2020). Hence, engaging with the sea from the land, such as when sea gazing, is worthy of further exploration as it may offer a more accessible and low-cost way of enjoying the well-being benefits of the sea.

## Viewing the sea and well-being

Although few studies have focused on the impact of engaging with the sea from the land, findings have suggested that simply *looking* at the sea may benefit health and well-being. Having a sea view has been associated with better mental and general health (Garrett et al., 2019; Nutsford et al., 2016); indeed Dempsey et al. (2018) found that the highest amount of sea view visibility was associated with a lower incidence of depression. Qualitative studies have touched on the experience and consequences of sea gazing within their broader findings about spending time at the coast or participating in activities in the sea. For example, participants have claimed that looking at the sea can help regulate their emotions and promote positive emotions of awe, joy, happiness, contentment and nostalgia (e.g., Jellard & Bell, 2021; Pearce et al., 2017; Severin et al., 2022). Participants have highlighted the calming, restorative nature of sea gazing, with looking at the sea believed to offer an undemanding escape from the stresses and responsibilities of modern life (e.g., Bell et al., 2015; Wright et al., 2024). Viewing the sea has also been claimed to facilitate transcendent experiences and a new perspective on one’s place in the wider universe (e.g., Jarratt & Sharpley, 2017). However, to the best of the authors’ knowledge, only one study to date has *focused* on exploring the experience of sea gazing from the land in any depth. In their Interpretative Phenomenological Analysis (IPA) study with women aged 30–50 years old, Hurdman and Kampman (2024) found sea gazing to be a captivating, embodied and multisensory experience, which strengthened participants’ enduring bond with the sea, and was believed to positively impact well-being. Nevertheless, this was a small idiographic study, and it did not seek to *explain* the relationship between sea gazing and well-being. Thus, there is scope for more robust exploration of the experience of sea gazing, and how it may relate to well-being, in order to better understand the potential of this activity as a positive health behaviour.

In summary, there is a growing body of evidence that spending time by, and in, blue spaces, especially the sea, is associated with benefits to psychological and physical well-being. Qualitative studies have begun to shed light on how specific types of engagement with the sea are experienced, and how they may be differentially related to well-being outcomes. However, to date, most researchers have focused on the well-being outcomes of activities involving immersion in or on the sea. Nevertheless, whilst the current evidence base is limited, research suggests that looking at the sea from the land may also offer benefits to well-being and is therefore worthy of further study. Better understanding the role sea gazing may play in the well-being of women in midlife may be especially pertinent given the aforementioned research highlighting the value of adopting positive health behaviours in midlife—and the paucity of well-being research among women in this life stage. In-depth qualitative methods may offer the required sensitivity to help us understand how sea gazing may be experienced and how it may relate to the well-being of women in midlife. In light of this, the present study took an *exploratory* rather than hypothesis-led approach to scientific enquiry and employed grounded theory methodology. This approach is particularly helpful when little is known about a topic and existing theories do not adequately explain a specific process or relationship. The overall goal of the study was to develop a substantive theory that explains the relationship between sea gazing from the land and well-being, situated in the context of women in midlife who live in coastal communities within the English counties of East and West Sussex. Four main research questions explored sea gazing as a dynamic process: (1) What motivates people to sea gaze? (2) What is the lived experience of sea gazing? (3) How does the experience of sea gazing relate to the experience of well-being? (4) What are the key personal and situational factors influencing this relationship?

## Methods

### Philosophical orientation and methodology

Grounded Theory (GT) is recognized as an insightful methodological approach when the research area under examination is not satisfactorily informed by pre-existing theory (Strauss & Corbin, 1998). Hence, given that the relationship between sea gazing and well-being is yet to be explained, GT was the methodology selected for the present study. GT has been described as a *total methodology* (Weed, 2009) that provides a set of principles for the entire research process and the outcome (i.e., a substantive theory). Several variants of GT, underpinned by different philosophical positions, have been proposed (for reviews, see Sebastian, 2019; Weed, 2017). To ensure methodological coherence, it has been argued that the specific variant of GT selected should reflect the researcher’s own philosophical beliefs (Holt & Tamminen, 2010). Accordingly, Corbin and Strauss’s (2015) variant was used in this study as their approach is consistent with the first researcher’s pragmatist and interactionist world view. Corbin and Strauss also view the researcher as not detached from the research process, but rather as engaged in the active interpretation of individual perspectives in order to develop a theoretical understanding. Thus, it is acknowledged that the substantive theory developed in the present report represents only one interpretation of the data, bounded by the specific contextual conditions in which it is situated.

### Participants and sampling

Fifteen female participants, aged 47–63 years (*M* = 53.87 years) were recruited to participate in the present study (see Table I for socio-demographic details). All participants were involved in stage one (semi-structured interviews) and of those, six also took part in stage two (submitting audio or written diaries). Criterion sampling was used to select participants able to provide detailed data relevant to the specific research topic (Patton, 2015). Hence, participants who identified as females in midlife (defined in the present study as between 45–64 years old) were recruited for stage one on the basis that they self-selected as spending time *looking out to sea from the land* at least once a week—in part because they believed it had an impact on their well-being. The nature and duration of their sea gazing experiences, and the meaning of the term *well-being*, were left unspecified. Thus, participants were able to impose their own interpretations of these criteria. Additionally, participants were required to live within the Brighton postcode area. This area lies on, and adjacent to, the South Coast of England, within the counties of East and West Sussex.

**Table I.** Participant socio-demographics (*N* = 15).

Variable	Category	n	%
Relationship Status	Single	5	33.3%
	Co-habiting	4	26.7%
	Married	5	33.3%
	Divorced	1	6.7%
Children	Yes	11	73.3%
	No	4	26.7%
Religion	No religion	10	66.7%
	Christian	4	26.7%
	Unspecified	1	6.7%
Ethnic Group	White	14	93.3%
	Multiple ethnicities	1	6.7%
National Identity	British	12	80.0%
	English	1	6.7%
	Scottish	1	6.7%
	Unspecified	1	6.7%
Employment Status	Employee	12	80.0%
	Self-employed	3	20.0%
Educational Qualification	Degree level or above	12	80.0%
	Another kind of qualification	3	20.0%

Consistent with one of the core principles of GT methodology, theoretical sampling was also introduced as soon as initial concepts were identified in the research data (Corbin & Strauss, 2015). Theoretical sampling seeks to identify the people, places, and incidents that allow the researcher to purposefully gather information about evolving theoretical ideas and concepts (Corbin & Strauss, 2015). Theoretical sampling continued throughout the research process until the concepts deemed important by the researcher were fully developed in terms of their properties and dimensions, and the perceived relationships between them were understood (i.e., theoretical saturation). To illustrate, early interviews comprised women who had lived by the coast for most of their lives, and their long-term residential proximity to the sea seemed to have been an important influence on its perceived well-being benefits. Subsequent interviews sampled women who had relocated back to the coast in more recent years, and women who had moved to live by the sea for the first time during the COVID-19 pandemic. This facilitated a deeper understanding of how the relationship between living by the sea and well-being may evolve over time.

### Recruitment

Initially, the first researcher used the social media platform WhatsApp to ask personal contacts to identify and refer other individuals within their social network who may fit the research criteria. Subsequently, the study was promoted on the Facebook pages of appropriate local community groups. As individuals possessing the key study criteria were deemed difficult to reach, a *snowball*, or referral, sampling approach was also employed, whereby participants selected for interview were asked to identify, and refer, other individuals who may fit the study criteria.

Prospective participants were sent an email that provided information about stage one of the study (details below). This email detailed the voluntary nature of the study, provided assurances regarding the confidentiality of all data and included a link to a short questionnaire and consent form, hosted by the Qualtrics XM survey tool. The questionnaire assessed participants’ eligibility to participate and collected voluntary socio-demographic data including age, gender, nationality, religion, education, relationship status and employment status. Participants who met the study criteria, and gave their informed consent (via completion of an online consent form) were contacted by the first researcher to arrange semi-structured interviews as detailed below.

### Data collection

Ethical approval for this study was obtained from the Sciences and Technology Cross-Schools Research Ethics Committee at the University of Sussex (reference ER/SLH43/1, ER/SLH43/2, ER/SLH43/3). The study adhered to the Declaration of Helsinki for research involving human participants. For GT studies, multiple methods of data collection have been recommended to enable methodological triangulation of data (Flick, 2019). In this study, data were collected from semi-structured interviews and audio and written diaries. The latter were supplemented with photographs taken at the time of sea gazing. In total, approximately 25 hours of interviews, and 26 diary entries, were transcribed and analysed by the first researcher (293 pages of single-spaced text). Information from the academic literature was used to make conceptual comparisons, enhance theoretical sensitivity, and stimulate analytic questions.

#### Stage one – semi-structured interviews

As the present study required the collection of rich, detailed, first-person accounts (Corbin & Strauss, 2015), the first researcher conducted online semi-structured interviews with 15 women aged 45–64 years. Interviews were 45–95 minutes in duration and audio-visually recorded using Zoom videoconferencing software. Online data collection methods have been shown to be comparable to in-person interview techniques with regard to building rapport, sharing deep personal experiences, interview duration, and participation rates (Jenner & Myers, 2019; Thunberg & Arnell, 2022). Video recording of interviews also facilitated the collection of non-verbal communications alongside verbal narratives and inflections. All interviews were manually transcribed, verbatim, by the first researcher. The interview questions addressed the aims of the study and explored: a) different experiences of sea gazing (e.g., “Is your experience of looking out to sea always the same?”, “If not, tell me more about your different experiences?”); b) causal conditions (e.g., “What influences your decision to look out to sea?”); c) perceived consequences (e.g., “What happens to you when you look out to sea?”); and d) contextual factors (e.g., “Is looking out to sea something you do on your own or with other people?”, “Why?”). Additional prompts were used throughout interviews to bring forth explanatory details of participant experiences (e.g., “More specifically, what did you think about?”,”What emotions did you experience?”). During each interview, the interview schedule was employed flexibly to ensure that key topics were discussed, whilst giving participants the freedom to raise pertinent subject matter. Consistent with the principle of theoretical sampling, the interview schedule was regularly updated to elicit further information on the concepts, categories and relationships identified in the data (Corbin & Strauss, 2015). For instance, in later interviews participants were asked more specifically about the *overall* role that the sea played in their lives (e.g., “Has the sea always played a role in your life?”, “Has this role changed at different times in your life?”). Later participants were also questioned directly about theoretical concepts identified in previous data to determine whether they held personal relevance (e.g., “Some people have talked about having a *connection* to the sea—does that idea mean anything to you?”).

Semi-structured interviews continued until substantive new information related to the developing theory ceased to be identified in the data. However, at this stage, the first researcher recognized that certain important theoretical concepts were yet to be fully understood due to reliance on *retrospective* data. Thus, the study moved to a second stage of data collection to facilitate access to everyday participant experiences in real-time.

#### Stage two – audio and written diaries

This stage involved participants recording a minimum of four diary entries, during, or shortly after, looking out to sea, over a four-week period. The participants were also asked to take photographs of the sea-view that accompanied each diary entry. Individuals were able to select their preferred mode of diary completion and submission as audio (voice notes or WhatsApp) or written (WhatsApp, SMS or email). The sample size was not pre-determined. Rather, following the approach of Karageorghis et al. (2018) and Thrower et al. (2016), participants who provided the most detailed and richest accounts in their earlier semi-structured interviews were initially recruited, with additional participants being added until theoretical saturation was reached. Between them, six participants provided 22 audio diary entries, 4 written diaries, and 28 sea-view photographs.

Participants were briefed orally, and in writing, about the stage two data collection aims and protocol; and given guidelines regarding the suggested focus of their diaries. Consistent with the theoretical sampling approach, participants were recruited at staggered intervals (approximately 1–2 weeks apart). This allowed briefing guidelines to continually evolve to ensure that data from diary entries filled outstanding gaps in the developing substantive theory (Corbin & Strauss, 2015). As an example, earlier participants were asked to record, in detail, how they felt emotionally, physically, and mentally *in the moment* as they looked out to sea. However, later participants were also asked to explain if, and how, they perceived that their experience *changed* over the duration of a single sea gazing experience.

Within one week of completing their diary entries, each participant took part in a second online semi-structured interview with the first researcher (40–60 minutes in duration). Individual interview schedules were tailored to allow each participant to clarify and expand upon their diary entries, whilst also aiding the purposeful gathering of information needed to clarify evolving theoretical concepts. Photographs submitted by individual participants were used, as appropriate, as a prompt during their follow-up interview. Each interview was audio-visually recorded using Zoom conferencing software and transcribed verbatim. Stage two data collection continued until no new information relevant to the developing substantive theory was identified in the data, that is, until theoretical saturation was reached (Corbin & Strauss, 2015).

### Data analysis

Within grounded theory (GT), data collection and analysis are viewed as interrelated processes to be carried out systematically and sequentially (Corbin & Strauss, 1990). Thus, the analysis of data was an iterative process that began as soon as the first data were collected and continued throughout the study (Corbin & Strauss, 2015). This ensured that developing ideas and concepts always guided subsequent data collection. Consistent with the principles of GT, the analysis of stage two data (diary entries and post-diary interviews) was informed by, and built upon, analysis of stage one semi-structured interview data. Throughout data analysis, the method of *constant comparison* was used to identify similarities and differences within, and between, participants’ accounts (Corbin & Strauss, 2015). Initially, raw data were compared with raw data, then raw data with ideas or *concepts* in the data, then concepts with concepts, and latterly, comparisons made between concepts and the literature. Comparisons were constant and ongoing throughout the analytical process to ensure that insights were grounded in all parts of the data (Corbin & Strauss, 1990). Written and audio memos were used throughout data collection and analysis to stimulate and record the analytic process and encourage reflexivity (Corbin & Strauss, 2015). In conjunction, diagramming was frequently used to conceptually visualize data, facilitate thinking at a more abstract level and communicate and discuss ideas, for example with *critical friends* (Stenhouse, 1975). Memos and diagrams are considered core analytic tools in GT (Corbin & Strauss, 2015) and were used to generate and develop concepts and categories (and their properties and dimensions), speculate on how they may be related, and identify gaps in knowledge that needed further exploration.

The process of analysis began with the first researcher re-reading each transcript to obtain an overall sense of the interview context. Initial, or *open coding* was then carried out, on a line-by-line basis, to identify concepts in the data that related to the research topic (Corbin & Strauss, 2015). These concepts were assigned labels, or *codes* e.g., “alleviating stress and anxiety” and “feeling part of the sea”. Once key concepts, including their properties and dimensions, were identified in the data, analysis moved to *axial coding* (Corbin & Strauss, 2015). In this stage of analysis, lower-level concepts pertaining to the same phenomenon were grouped into higher-level concepts, or *categories*, and potential relationships between those categories were identified (Corbin & Strauss, 2015). For example, concepts describing different motivations for sea gazing experiences were initially grouped under the category “I know it will always make me feel better.” The iterative process of open and axial coding, and constant comparison, continued within and between transcripts throughout data collection.

The final stage of analysis was *theoretical integration* (Corbin & Strauss, 2015) during which a *core category*, or central phenomenon, of the study was identified, links between the core category and other categories were established, and a preliminary theoretical model was developed. During the early stages of theoretical integration, the developing theory was perceived as lacking explanatory power. The first researcher recognized that reliance on retrospective data from interviews limited full development of certain categories identified as important to the overall theory. For example, in interviews, participants were able to give rich, detailed information about particularly memorable sea gazing experiences in times of trauma, such as the death of someone close. They were also able to recount, in some detail, the enduring, and multifaceted role that the sea played in their lives and how this had evolved over time. However, they were less able to explain their *specific* reasons for more everyday sea-gazing occasions, or the exact nature of their sensory, cognitive and emotional experiences *in the moment* as they looked out to sea. Hence, it was at that point in the analytic process that the first researcher identified the need to introduce a second method of data collection and analysis (i.e., diaries and post-diary interviews). The aim was to capture information related to *everyday* motivations and *in the moment* experiences and outcomes. Analysing data from these diary entries subsequently revealed, for example, that during specific sea gazing events, participants typically switched, sometimes numerous times, between focusing on the external world of the vista and their internal world of thoughts, emotions and bodily sensations.

Data analysis ceased once six participants had completed their diary submissions, at which point all the data had been integrated into a satisfactorily dense and explanatory theory.

### Methodological rigour and trustworthiness

In addition to the previously described methods (i.e., theoretical sampling, iterative data collection and analysis, and constant comparisons), several further strategies were employed to promote rigour and trustworthiness during the research process. As previously discussed, the first researcher used memos and diagrams throughout the data collection and analysis process. Written memos (*n* = 82) ranged from a few lines to three pages of single-spaced text in length, and audio memos (*n* = 74) were between 1–10 minutes long. Second, other members of the research team, and the wider academic community of the researchers’ institution, regularly took the role of *critical friends* in terms of challenging interpretations and providing different perspectives through which to view the data (Costa & Kallick, 1993). The study methods, developing concepts and theories were discussed and presented formally and informally at various stages of the research process. This provided further methodological and theoretical challenge for the first researcher. Third, theoretical credibility was enhanced by eliciting *member reflections* on the developing theory (Olmos-Vega et al., 2023). Five participants volunteered to reflect and build on the first researcher’s interpretation of their data in online Zoom discussions. In this way, member reflections helped generate additional data and insights and were used to refine the final theoretical framework. Fourth, as the first researcher is a coastal-dwelling woman in midlife, and a regular sea gazer, she kept a reflexive written and audio journal and field diary throughout the study. This allowed her to record preconceived ideas and potential biases in relation to the research questions and participant data; and reflect upon her responses to participant interactions and their narratives. Recognising that she was part of, rather than independent of, the research process (Corbin & Strauss, 2015), the first researcher also used the journal to understand when, and how, her subjective perspectives may impact study outcomes. Finally, the quality of the resultant substantive theory was judged based on the concepts of fit, work, relevance and modifiability (Corbin & Strauss, 2015).

### The use of literature

In contrast to traditional GT (Glaser & Strauss, 1967), Corbin and Strauss (2015) encourage researchers to acquire some knowledge of the extant literature to develop a strong study rationale. Thus, prior to commencing the present study, the first researcher gained a broad understanding of the academic literature related to *blue spaces and well-being*. This identified a lack of research specifically exploring the relationship between engaging with the sea from the land and well-being. Within this field, women in midlife we also identified as an under-researched population. Subsequently, at appropriate points during data collection, data analysis and theory development, the extant literature was used to ensure theoretical sensitivity (Corbin & Strauss, 2015). Relevant theories and constructs from the literature were used to explore concepts identified in the data, compose interview questions, and ensure that categories were fully developed in terms of their properties and dimensions.

## Results

**Figure 1.**
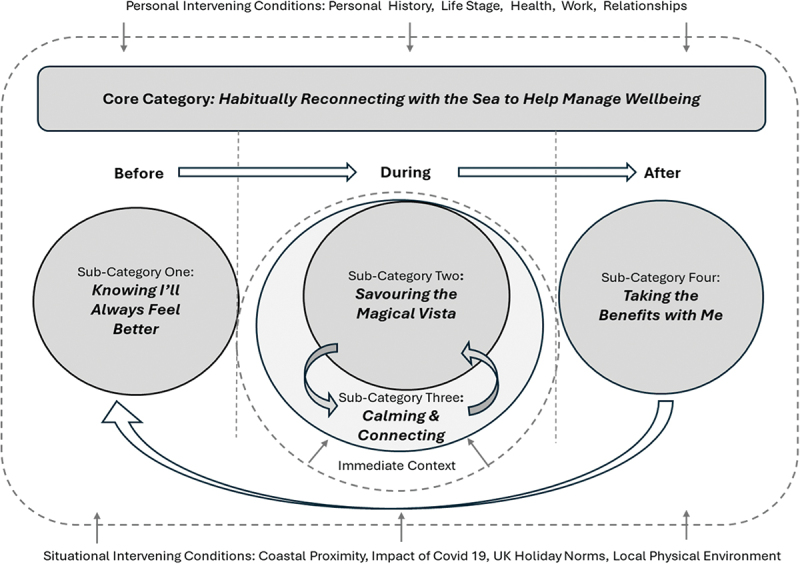
A substantive theory of the theoretical process through which sea gazing impacts the well-being of women in midlife.

Following the analysis of interview and diary data, Figure 1 is a diagrammatic representation of the theoretical process through which sea gazing is proposed to impact the well-being of women in midlife. This substantive theory is constructed around a core category of *habitually reconnecting with the sea to help manage well-being*. The core category is underpinned by four sub-categories: *knowing I’ll always feel better; savouring the magical vista; calming and connecting;* and *taking the benefits with me*. The proposed theory is first summarized, and then the core category and four sub-categories are explained in greater detail. The relationships between categories, and important contextual factors, are also discussed within relevant sections. Participant pseudonyms are used throughout to preserve anonymity.

The immediate context includes prevailing weather and sea conditions, current state of well-being, social context, type of exposure (indoor or outdoor), time of day, and activity level (e.g., sitting, walking, running)

### Summary of substantive theory

The central idea, or core category, of the proposed substantive theory suggests that the coastal-dwelling women in the present study habitually used sea gazing to help them manage their well-being. Sea gazing was used by participants in this way because it was believed to reliably maintain and enhance their experience of connectedness to the sea, thereby promoting well-being outcomes. This was a self-care practice, perpetuated because, based on previous experience, individuals were drawn to the sea knowing that it would *always* make them feel better (sub-category one). Whilst sea gazing occasions may have been triggered by different events (e.g., trauma, pleasant weather) and for different purposes (e.g., coping, mood-enhancement, seeking time alone), they were all underpinned by the belief that sea gazing would unfailingly lead to well-being benefits.

In this study, the act of sea gazing was shown to be a proactive *savouring* experience during which individuals regularly chose to be immersed in the magic and majesty of the sea vista (sub-category two). This was a multisensory and embodied experience that mindfully captivated participants in the pleasurable present moment and promoted an emotional uplift. In turn, this savouring experience was perceived to facilitate a calming of the mind and body, positive introspection, and transcendence beyond self-boundaries (sub-category three). As a direct consequence of this savouring, calming and connecting experience, individuals felt that their well-being had been restored, reset, and/or enhanced as they returned to their daily lives (sub-category four). However, the specific well-being benefits that lingered after each sea gazing event diminished over time. Hence, individuals were strongly motivated to habitually engage in this pleasurable activity as part of their ongoing well-being management.

Overall, the proposed theory explains a self-reinforcing, cyclical process during which each sea gazing event facilitates enhanced connectedness to the sea and positive well-being outcomes. In turn, habitual experience of these benefits reinforces the role of sea gazing as a reliable self-care practice, which prompts further sea gazing events.

#### Core category: habitually reconnecting with the sea to help manage well-being

As women in midlife, participants in the present study were typically experiencing a number of significant life events including the menopausal transition, chronic illness, career changes, and caring for elderly parents. As a result, they had numerous demands for their time and energy. In light of this, they routinely took time out of their busy lives to *reconnect* to the sea by gazing onto the vista. This self-motivated practice was perceived to increase their feelings of connectedness to the sea, and to reliably promote positive well-being outcomes. In this way, participants habitually used the sea as a resource to help them manage their mental, emotional and physical well-being. In the present study, key properties of the core category were conceptualized as: *connectedness to the sea; self-care practice;* and *reliability.*

All participants demonstrated a powerful subjective sense of *connectedness to the sea*, that was experienced across cognitive, emotional—and for some, spiritual—dimensions. Notably, *sea* connectedness was experienced as distinct from how participants related to other parts of the natural world. For example, Veronica explained how she connected to the sea as “unique in nature”:

The sea is the mother … .I see it as a feminine power, I feel a connection in that way. I do really. I do really feel I really feel the feminine force of the sea.

For most, feelings of connectedness to the sea had begun in childhood and had intensified, and become more multifaceted, over time. The United Kingdom norm of regular *seaside holidays* meant that the sea was typically associated with “happy childhood memories” (Diana). For most participants, these early memories seemed to have laid the foundation for the strength of their connectedness to the sea in the present:

It’s always been a connection … childhood memories, family memories… it’s always been things that you kind of associate with good memories … I think maybe it’s just something that builds over your lifetime that you are presented with those opportunities to build an affinity with the sea. (Reba)

For some, their strong sense of connectedness to the sea had developed later in life. For example, the COVID-19 pandemic represented a watershed period, highlighting the important role the sea played in their lives and giving some participants the impetus to fulfil their dream of relocating to the coast.

In this study, sea connectedness was experienced, in part, as an integration of the sea into participants’ emotional and cognitive representations of the self. Notably, participant accounts suggested that both the sea as a “living entity”, *and* sea gazing as a behaviour, were experienced as part of their identity, and as part of their lives. For example, Louise did not seem to differentiate between incorporating the sea per se, and her daily sea gazing practice, into her conception of self:

I do feel I’m part of it. And it’s part of me. It’s part of my make up now is that it’s yeah that’s part of my daily routine. Yeah. Yeah. And all my friends know it about me and my partner knows it about me. And the kids know it about me. It’s part of my identity. (Louise)

When participants discussed their connectedness to the sea, they often characterized it as a multifaceted *relationship*. Indeed, participants often expressed their deep and unconditional love for the sea. The sea was typically viewed as an *active* contributor to the relationship and, as such, supported the emotional, and for some spiritual, needs of participants. The relationship between the sea and self was generally viewed as unconditional, and unequal, as participants described being able to take whatever they wanted and needed from the sea without having to offer anything in return. This was evident when Reba spoke of how her relationship with the sea was unlike any other in her life:

It is that constant in your life that doesn’t ever want anything back from me, whereas obviously, you know, friends, relationships, you know all those relationships are, you know, it’s all about reciprocation, isn’t it? You give and take. And whereas you can be quite selfish with the sea. Take what you need from it.

Participants often struggled to understand and verbalize *how* the sea—as a non-human entity—was able to help them in ways that perhaps their human relationships could not. Hence, when attempting to rationalize the unique role the sea played in their lives, they often relied on anthropomorphic metaphors. The sea was regularly described as like a “mother,” “good friend,” “therapist” or “lover”; and imbued with the positive qualities associated with those roles. For example, Clover described her “love affair with the water that sustains me,” whilst Flo could “turn to the sea, and it’s almost like a therapy. It’s a therapeutic experience.” For Reba, the sea was like a “best friend” she could rely on to always make her feel better.

Knowing that reconnecting to the sea would always benefit well-being was a powerful motivator for participants to continue to regularly sea gaze. Hence, the second property within the core category was conceptualized as *self-care practice*, as participants typically used sea gazing as a positive health and well-being behaviour. Whereas most of their time and energy were focused on the needs of others, sea gazing represented one of the few (sometimes only) ways they focused on their own well-being needs. As such, they ensured that sea gazing was incorporated into their regular routine—whether it was cycling or walking to work via the seafront, dog-walking on the beach or stretching whilst looking out to sea after a run. As Louise explained:

… it’s literally just become part of my daily routine that I need to be by the sea … I think that’s when I started like making it part of my life, because I knew it made me feel so good every time I was there. … it’s my daily kind of thing that’s gonna make my day good. It’s trying to grab a bit of that to bring me back into myself.

Participants’ use of the sea as a self-care practice was supported by their belief in the *reliability* of the sea that was “always there for me” (Emmie). Indeed, the sea “felt like home” (Flo) and represented an important anchor in their lives. The sea held the reassuring promise of permanence, as it always had been in existence, and always would be, long after they were gone. In light of this, sea gazing was used as their coping mechanism of choice for “self-healing” (Jayne). For example, Sapphire recounted how the sea reliably helped her manage her anxiety:

Just knowing it’s there and knowing how it’s helped in the past … and I know it will always help. It will always yeah help in the future … . I’ve now got an understanding that this can help me, regularly, to cope with it.

#### Sub-category one: knowing I’ll always feel better

As a consequence of past experiences of looking out to sea, participants anticipated that *every* sea gazing event would lead to positive well-being outcomes. Whatever their mental, emotional, and/or physical state prior to sea gazing, and in whatever context, participants believed that sea gazing would *always* make them feel better. The continuum was one-directional as participants always anticipated that sea gazing would lead to positive well-being outcomes. What changed was the nature and degree of benefits they expected as a consequence of specific sea gazing occasions. This category was developed around three interrelated properties: *drawn to the sea; different motivational drives;* and *time and space for me.*

Participants felt a powerful emotional, and sometimes almost gravitational, draw towards the sea that seemed to defy rational explanation. Their desire to be close to the sea was experienced at different levels of intensity and was perceived as either an acquired or an “instinctive” drive. For example, Tallulah believed that her need to be close to the water was innate and:

… like a physical pull. Like it feels like my heart is pulled towards the sea… It comes from something really deep inside me, like now I can kind of experience it now as something deep in my chest that feels activated by the thought of sitting by the sea.

In contrast, Emmie believed that her need to be by the sea developed from early learned behaviour:

It was acquired, but it was acquired before I realized it, because it was right from the very get go of being very small.

When travelling away from the coast, or having moved inland for periods of their lives, participants described experiencing a profound sense of loss, and feeling “trapped” or “claustrophobic”. They always felt drawn back to the sea and found that the idea of a permanent move away from the coast almost unimaginable.

When reflecting upon the reasons that triggered specific sea gazing events, participants recognized that they were prompted by *different motivational drives*. At one end of the range of motivational drives was the desire to seek, or enhance, feelings of pleasure. For example, Clover explained that when in a positive mood on a warm, sunny day, she decided to sea gaze because she:

….felt that delight in me and I thought, I’m consciously I’m going to go and sit by the sea because it will just amplify the delight.

Hence, when seeking pleasure, the motivation to sea gaze was experienced as more of a *want* than *need*. In contrast, participants recalled feeling a powerful *need* to sea gaze in response to trauma and pain. The sea represented the one place they knew would help them heal. Tallulah explained how she was drawn to the sea when facing the trauma of working in a hospital during the pandemic:

In the COVID times, in the lockdown, when we were working night shifts in the hospital, and it was all very stressful, and people were dying, then I would go down, particularly if I’d worked a night shift, I would go down first thing in the morning … I would just go and sit by the sea.

Between these two motivational extremes, participants also anticipated that sea gazing would help ameliorate the impact of everyday stressors. As Christine explained:

So if I wasn’t having a great day, I went down, and you know, sat down by the sea - and I’m not talking about major life traumas, I’m talking about just things that kind of throw, throw me out of kilter a bit - and then I’m realizing, even in that moment … it can kind of fix those things.

Importantly, whatever motivated specific sea gazing events, choosing this action over any other was underpinned by the belief that sea gazing was one thing guaranteed to make them feel better. Participants often favourably compared sea gazing to other well-being practices—such as spending time in green spaces, taking a long bath, or watching a favourite movie. In all cases, sea gazing was cited as the most effective and reliable action for boosting well-being. Indeed, the sea seemed to play an unparalleled role in participants’ well-being management through facilitating a unique set of benefits. As Reba stated with conviction:

What would I do if I didn’t have that? Where would I go? What would have an impact on my ability to actually be able to problem solve some things, or, you know, just to go and self-sooth, or feel better, or have those moments where you could just kind of empty your brain. You know? Where would I go to do that? How would I do that if I wasn’t near the sea?

Whether driven by want or need, participants consciously engaged in sea gazing as a solo pursuit. They knew that being alone while looking out to sea would give them the literal and metaphorical space they struggled to find in their busy lives. In this way, sea gazing created much needed *time and space for me*, and provided a sense of freedom and escape from their stresses and responsibilities:

… it’s a way of just having that little bit of time where you’re not answerable to anyone at that point, or you don’t feel the need to help, or have to do stuff. (Dora)

In general, because participants were aware of the importance—yet rarity—of time for themselves, they anticipated that sea gazing alone would lead to greater well-being benefits than sea gazing alongside close others, or in a busy social context.

#### Sub-category two: savouring the magical vista

Whatever the given reason for sea gazing, this activity was always explained as a pleasurable *savouring* experience. Participants consistently recalled being captivated by, and immersed in, the sea vista; held in the present moment while they lingered on all they could see, hear, smell and feel. Experiences of being mindfully and sensorially engaged in appreciating the “magic,” “majesty” and “magnitude” of the vista were recounted in rich detail. Hence, the sub-category of *savouring the magical vista* was developed around four interrelated properties: *multisensory embodiment; immersed in the moment; captivated by beauty and majesty;* and *emotional uplift*.

Savouring the vista within the context of the present study was experienced, in part, as *multisensory embodiment*. Participants readily described the engagement of all their senses while sea gazing:

I think it is about all the senses to me with the sea … it’s that immersing myself in what I can hear and see and smell, and whatever, and that I think that is it. I think that actually just helps me to be more present. (Lily)

You’ve got the visual aspect, but also you’ve got the noise as well like, you know, if you’re sitting on the beach, and it’s quite calm, you’ll just have so gentle kind of lapping of the waves on the on the beach, or if it’s bit more stormy, it’s just comes more of a crash. You’ve got the smell of the sea as well which sort of really fresh and kind of “ozoney” …. and it’s also tactile as well. (Anne)

However, all the women in this study were sighted, and the pleasurable *visual* experience of sea gazing seemed to be the most prominent aspect of their sensory engagement. This was evidenced by the detail with which they described their visual experiences, and the extent to which seeing the vast, seemingly never-ending vista facilitated positive cognitions, emotions, and bodily sensations. In one of her diaries, Clover explained:

I’m really, really caught by the sparkling light on the water … .I’m now just raising my gaze towards the horizon and noticing just how far back into the distance I can see the flashing, sparkling reflections of the sunlight. I can see the quality of it. And feel the movement of it. And it really, really, raises my spirits. And I feel happy.

The enormous scale and openness of the vista, along with the repetitive, rhythmic movement of the water, was generally considered to be unique in nature. The constant ebb and flow of the waves were not only visually captivating but also experienced by participants in an embodied sense:

It’s something about the frequency of the waves as well, like the kind of variation and frequency of the waves that feels like it soothes something in my nervous system. (Talullah)

The sea was perceived as a living, breathing entity, and participants metaphorically described ingesting the sea as they slowed their own breathing pattern to attune to the rhythm of the sea. Overall, the sounds, smells, touch, and even taste of the sea, all combined with visual cues to create an immersive mind-body experience.

When looking out onto the vista, participants became *immersed in the moment* and recalled being able to fully attend to, and appreciate, all that they could sense and feel. Sea gazing was often likened to a mindfulness or meditation experience but generally recalled as a less effortful way to focus on the present:

But it’s just almost like mindfulness …. I’m not very good at that, anyway. So sit me down and close my eyes and tell me to meditate. I was like “woo, that’s not so good”. I’m so distracted sometimes, but the sea has that effect on me. You can’t control it, can you? (Dora)

Unlike mindfulness and meditation practices (which focus on observing and letting go of good, bad, or neutral thoughts), looking out to sea was shown to primarily facilitate lingering on pleasurable thoughts and sensations. As a consequence of being able to effortlessly savour the present moment, participants were able to release themselves from focusing on the stresses and anxieties in their lives:

I think it tends to put me in that moment, rather than thinking about stuff that you know, may or may not have annoyed me, or you know upset me, or whatever. (Christine)

While sea gazing, participants were able to savour the present because they were *captivated by the beauty and majesty* of the vista before them. Their narratives were peppered with evocative adjectives such as “mesmerising,” “hypnotic” and “magical”. In particular, the vast scale of the sea vista never failed to amaze participants and hold their attention:

But the thing with me always is the size – it’s massive! It goes on and on and on as far as you can see! … I mean to me, nothing’s bigger, I don’t think. Is anything bigger … It’s massive, it’s huge …. it’s alive as well. It’s not just like a massive big building over there …. it’s alive, it’s emotional, it’s playful. (Felicity)

The sea enthralled and intrigued—in part because it was ever-changing. As such, prevailing weather and sea conditions had a significant impact on how each sea gazing event was experienced. The sea could shift from possessing a calm and magical beauty on one day, to being a raging, powerful and majestic cauldron on another. Yet it *always* captivated attention. For example, in interview Sapphire contrasted the engrossing nature of two of her sea gazing experiences, first describing a calm sea on a balmy day:

The sun was glistening on the sea….I think that’s got to be the best outlook. When you see that shimmer on the sea, on the water … it was just a lovely light, and then that, really you know, drew me in, and I couldn’t stop looking at it …

Then a rough sea on a stormy day:

On the, yeah, the wilder, the wilder days, it’s still magical, I think, because it is the powerful force isn’t it? It is just that powerful force. It just it just grabs you, and it almost says, “Look at me!”

Notably, regardless of the weather and sea conditions, sea gazing was always perceived to promote an immediate *emotional uplift* for women in this study. Indeed, savouring the vista could facilitate numerous positive emotions in the moment. These ranged from joy, contentment, happiness, gratitude and serenity, to awe, wonder, excitement and nostalgia. Influenced by contextual factors, the nature and intensity of these emotions varied by event, and by individual, but participants *always* experienced a positive mood shift while sea gazing. As Emmie simply stated:

… seeing that beauty just makes you feel happier.

#### Sub-category three: calming and connecting

The experience of savouring the magical vista was perceived by participants to facilitate numerous interconnected emotional, cognitive and physiological processes. In the present theory these are summarized by three key properties: *calming mind and body; introspection;* and *self-transcendence.*

For all participants, sea gazing reliably promoted feelings of calm, peace and serenity in the mind and body. This sense of *inner calm* was experienced *during* each sea gazing event and was perceived to linger (with varying strength and duration) as participants returned to their daily lives. Perhaps counterintuitively, participants claimed that sea gazing would always *initially* promote a sense of calm, regardless of prevailing weather and sea conditions. For example, when asked if different sea conditions led to wholly different outcomes in the moment, Dora replied “’No … I still get the calmness and clarity. Yeah, even when it’s really stormy.” And in one of her diaries Flo recalled feeling “ … .calm. Although the lashing waves are covering me in spray there is a tranquillity even in this turmoil.”

Participant accounts suggested that this sense of inner calm was facilitated by, and was a consequence of, internal processes related to mental, emotional and physiological release and relaxation. Regardless of the sea and weather conditions, looking out to sea was generally believed to effortlessly empty the mind of the practicalities, stresses and anxieties of life. Clearing this unwanted mental “clutter” was perceived to promote mental clarity for participants. As Veronica explained:

It’s kind of like my troubles diffuse in that moment, and it gives me like clarity, calmness of mind.

In addition to a sense of mental release, participants also experienced the release of unwanted emotions. The nature of the emotions from which participants disengaged varied—from those associated with grief and trauma to those arising from daily annoyances—but they were all aversive. Participants also frequently reported experiences of *physiological* release while sea gazing, as looking out to sea was perceived to reduce their heart rate, lessen stomach churning, deepen their breathing and relieve tension. Clover explained this in one of her diaries:

I’m yeah just noticing the feeling of relaxation coming through my body. Kind of just easing the tension. Across my shoulders, around my chest.

The release and relaxation experienced by participants while sea gazing created a sense of *space* in mind and body. This internal space, together with the external space achieved by sea gazing alone, seemed to give participants the capacity to be introspective, and to work through unresolved thoughts and feelings. For example, Lily explained how sea gazing encouraged her to prioritize rather than ruminate:

… it kind of gives you space … when I’m down there I can problem solve a little bit more, I can kind of I can kind of sort of classify my worries and think like “right, that one, I really don’t have to worry about that”. … It just gives me an opportunity to put things in order a little bit, and I think that’s probably where, why my tension starts to reduce and (I get) that sense of calm.

Importantly, sea gazing was shown to facilitate *positive* introspection as participants seemed able to reflect and gain clarity with an adaptive mindset, even if the subject matter itself was challenging or emotionally charged. However, not all participants looked inward while gazing at the sea. Whilst some participants repeatedly shifted their attention between their internal and external world, others claimed to rarely, or never, focus on anything other than the vista before them. As Emmie explained:

I just I don’t think when I’m looking out to sea, I don’t think I don’t think about anything that that’s going on in my life that isn’t on the sea … . it just gives me a real sense of calm and I lose all of this stress. … . afterwards I can get back to work, the exasperation feeling has gone and I just feel calmer and can see things clearly.

While sea gazing, participants not only engaged in introspection, but also related to the world beyond self-boundaries. Savouring the magical vista was shown to facilitate self-transcendent thoughts and feelings as participants related to the sea as “greater than the self,” both literally and metaphorically. Perceiving the sea as a “greater power” promoted feelings of comfort, reassurance and safety, and self-transcendent emotions of awe, wonder, majesty and amazement. For many participants, the feelings of connectedness they experienced while sea gazing had a spiritual dimension, with the sea perceived as “magical,” “mystical” and “otherworldly.” Importantly, connecting with an entity they perceived as greater than themselves as a mere human gave participants perspective as to their relative unimportance “in the grand scheme of things” (Felicity). Perceiving themselves as insignificant was experienced positively in this context because it served to diminish the importance of their problems and anxieties, rather than themselves as individuals:

… there’s something bigger than you, and the situation out there, and that’s what puts into perspective that we’re in this, this is nature, and this is huge … It’s like just going on forever. Just helps me re-evaluate what might be stressing me out. (Louise)

Recalling experiences of self-transcendence, participants described feeling a sense of “oneness” with the sea, the natural world and the universe beyond as they gazed at the vista. Diana tried to explain her experience of interconnectedness while sea gazing:

Like you’re just like part of everything … it just kind of like reminds you that you’re part of nature as opposed to, just like, you know, in your head, fretting about stuff, being a little drone bee, or wherever, you know, working for somebody else. You’re like “no, I’m not. I’m actually, you know, I’m part of this. I’m part of the universe”. (Diana)

Typically, participants experienced calmness, self-transcendence and introspection in an interconnected and cyclical manner while sea gazing. Diary entries in particular revealed how individuals moved back and forth between looking *into* the self, and connecting *beyond* the self, while experiencing feelings of inner calm. This cycle was often repeated multiple times during a single sea gazing event.

#### Sub-category four: taking the benefits with me

After the event, some or all of the benefits participants experienced while sea gazing were perceived to remain with them as they returned to their daily lives. Typically, participants most readily described the lasting impact of sea gazing as an improvement in their *overall* sense of well-being. However, analysis of interview and diary data showed that global well-being improvements were further characterized by participants as having properties of *restoration*, *reset* and *enhancement.*

Participants’ experiences of well-being *restoration* ranged from mitigating the impact of daily stresses and anxieties, to healing following major trauma and psychological and/or physical pain. For example, Veronica recalled how the sea helped her heal after experiencing a miscarriage:

I was feeling so lost … that’s kind of like, maybe that’s where it started, feeling this connection with the sea as a healing power.

Experiencing release and relaxation while sea gazing was believed to restore well-being by facilitating stress reduction. Whether stress and anxiety were experienced as chronic or acute, looking out to sea was claimed to be a “wonderful antidote to anxiety” (Clover) and often used by participants as their regular “coping mechanism” of choice. Sea gazing was also perceived to help restore mental and physical well-being following an illness. For example, in one of her diaries, Flo recalled feeling “pretty crappy, feeling sorry for myself and aching and rotten physically drained and mentally like a woolly zombie.” However, immediately after sea gazing she was “awake and feeling blessed to be able to be here. I start to smile and feel an inner happiness I’m unable to achieve anywhere else.”

Participants also used sea gazing to *reset* their well-being. Whether or not they were conscious of specific health deficits, looking out to sea was likened to activating a “restart button” (Sapphire), helping to return participants to their *set point*, or baseline level of positive well-being. In this way, sea gazing was shown to “set me up for the day” (Emmie), leaving participants feeling ready to tackle the practicalities, responsibilities and stresses they anticipated encountering. And knowing that the sea could help them “recharge the battery” (Sapphire), participants consciously made sea gazing part of their routine:

So I feel like I’m arming myself if you like. I’ve taken those deep breaths. I’ve looked out. It’s made me happy. It’s made me glad to be alive, made me really appreciate where I live, and I will do it again tomorrow, and so everything will be alright. (Louise)

For those with a sea-view, looking out to the sea could be used multiple times a day for well-being maintenance. As Dora said with a smile: “I’m lucky enough to say that I can reset at any point in the day if I wanted to.”

Sea gazing was also believed to *enhance* the emotional, mental and physical well-being of participants. As previously noted, savouring the sea was shown to promote numerous positive emotions in the moment. Notably, however, those emotions, and the accompanying uplift in overall mood, generally remained with participants as they returned to their daily lives. Clover and Anne’s emotional experiences were representative of many:

How lucky am I to have this available to me? So I can kind of go for that walk and stare at sea and go home and feel happier and more joyful afterwards than when I started. (Clover)

… it is definitely a kind of mood boosting sort of experience … .I come away from it I feel a lot lighter. (Anne)

Participants also experienced enhancements in their mental well-being after sea gazing. For many, clearing and calming the mind and body, and engaging in positive introspection, whilst sea gazing bought clarity and perspective to problem solving and promoted creative thinking. For example, after gazing at a particularly rough sea, Emmie recalled feeling:

… energized to be able to, you know, compartmentalize all that, and then see my way through the trees to actually sort out the issues.

Whilst sea gazing was always perceived to positively impact well-being, the specific nature and duration of benefits varied between individuals and events. The environmental context (e.g., weather and sea conditions), and personal factors (e.g., current state of well-being, time of day) influenced how participants felt after each sea gazing event. For example, Flo described how on one occasion “the huge lashing waves over the sea wall defences” had left her feeling “invigorated, alive and awake”. By contrast, the following day the sea was “ … glistening like silver, dancing around. It was exciting, new and different to yesterday” which instead left her feeling “peaceful and serene.”

Importantly, when participants left the sea and returned to their daily lives, whatever benefits they took with them were generally perceived to diminish over time: “You know, like drugs have a half-life or something. In an hour or a week, it’s gone” (Clover). Hence, the drive to keep “topping up” the benefits of sea gazing was shown to be a primary motivator for participants’ habitual use of this activity to help them manage their well-being.

## Discussion

The aim of the present study was to develop an explanatory theory of the relationship between sea gazing from the land and well-being for coastal-dwelling women in midlife. The resultant substantive theory explains a self-reinforcing, cyclical process in which sea gazing is used as a habitual, and unparalleled, self-care practice that plays an important role in overall well-being management. Whilst results showed that sea gazing occasions were triggered by different events, and intended for different purposes, they were all underpinned by the belief that sea gazing from the land would reliably promote well-being benefits. Iterative analysis of in-depth retrospective and real-time participant accounts revealed sea gazing to be a proactive savouring experience that perpetuated, and enhanced, participants’ emotional, cognitive and spiritual connectedness to the sea. Proactively savouring the sea vista was perceived to facilitate physiological, psychological and emotional release and relaxation, and so effortlessly calm mind and body. Consequently, participants returned to their daily lives perceiving that their well-being had been restored, reset or enhanced. Notably, whatever the well-being state of participants’ immediately prior to sea gazing, this simple activity was claimed to *always* lead to an improvement in overall well-being. Whilst the specific nature and intensity of well-being benefits experienced on each occasion were influenced by a range of contextual factors—in particular, the prevailing weather and sea conditions and immediate social context—participants believed that sea gazing could always be relied upon as a positive health and well-being behaviour.

The proposed substantive theory suggests that to gain well-being benefits from engaging with the sea it may not be necessary for individuals to be immersed in, or on, the water. Rather, the results indicate that some people reliably gain significant benefits from regularly gazing at the sea while remaining on land. These findings corroborate, and build upon, evidence from Hurdman and Kampman (2024) that regularly looking out to sea from the land was a mindful, multisensory and embodied experience, perceived to restore and enhance well-being for the women in their study. Findings are also consistent with the growing body of literature demonstrating that living by, and visiting, the coast is associated with higher subjective well-being, reduced mental distress and better self-reported general health and well-being (Bell et al., 2015; Dempsey et al., 2018; Gascon et al., 2017; Nutsford et al., 2016; White et al., 2020, 2021). Yet, to date, qualitative researchers in the “blue space and well-being” field have often focused on the experience, and benefits, of activities requiring immersion in, or on, the water, such as swimming, surfing and sailing (Britton & Foley, 2021; Britton et al., 2020). As such, the present in-depth study offers new insights into how sea gazing from the land may be experienced as similar, and as distinct, from pursuits requiring sea-immersion. Notably, many of the experiential and well-being themes emerging from sea-immersion studies are consistent with the findings of the present research. For example, both sea gazing and sea-immersion activities have been explained as emotional, mindful and embodied experiences that build connectedness with the natural world, change and expand participants’ perspectives, provide respite from daily challenges and anxieties, promote a range of positive emotions and facilitate a sense of improved overall well-being (e.g., Britton & Foley, 2021; Britton et al., 2020; Denton & Aranda, 2020; Foley, 2017; Murray & Fox, 2021; Olive & Wheaton, 2021). Thus, evidence suggests that sea gazing from the land may offer many of the same benefits as engaging in activities in or on the sea.

Nevertheless, the present study highlights differences regarding the experience and consequences of engaging with the sea from the land compared to being immersed within it. For example, participants in sea-immersion studies have often emphasized the social and physical aspects of taking part in pursuits such as sea swimming, surfing and sailing. Participant accounts have highlighted the *doing* of these immersive activities, generally within a social context, and have described the sea, in part, as the *enabler* of the activity in which they are engaged (e.g., Bates & Moles, 2021; Costello et al., 2019; Denton & Aranda, 2020; Foley et al., 2019; Lisahunter & Stoodley, 2021; Murray & Fox, 2021; Pipere et al., 2020). In contrast, the present study explains sea gazing as a solitary, *“just being”* experience in which individuals are captivated by the intrinsic beauty and majesty of sea vista itself, rather than focusing on their own behaviours. Indeed, participants in the present study often engaged in sea gazing while they were sitting or standing still along the shoreline or enjoying their residential sea-view. Researchers have also recognized that whilst sea-immersion activities are associated with a range of health and well-being benefits, they may have limited appeal and face barriers to participation including: a lack of skills and knowledge, limited physical abilities, financial cost, and a lack of opportunity (Ford & Brown, 2006; Jackson, 1983; Sailors & Weaving, 2024; Sotiriadou et al., 2020). In contrast, for individuals living with easy access to the coast, this study suggests that sea gazing from the land may offer a less costly, lower-risk and more accessible way to gain well-being benefits from engaging with their coastal locale.

However, it is acknowledged that the present findings are based on the experiences of a relatively small sample of women in midlife who were already regular sea gazers. Researchers have yet to understand whether sea gazing has wider potential as a positive well-being behaviour for coastal-dwelling midlife women in general. This presents an opportunity for researchers to explore more broadly the beliefs, attitudes, and behaviours of midlife women who do and do not currently engage in sea gazing, to better understand the well-being outcomes they derive or anticipate from it. To confirm and extend the findings of this research, future studies should include larger samples from a wider range of geographic locations and diverse ethnic, religious, and cultural backgrounds. Whilst sea gazing may be a simple, low-cost, and relatively accessible way to engage with the sea, it may not appeal—or offer benefits—for all. Hence, clarifying our understanding of how, when, and for whom sea gazing from the land may promote specific well-being outcomes would help determine the potential of this activity to play a role in proactive well-being management for women in midlife.

The proposed theory suggests that the nature of sea gazing as a *proactive savouring* experience may be an important factor linking this activity with well-being benefits. In the present study, sea gazing was construed as a proactive savouring process because participants regularly sought opportunities to be mindfully captivated, and immersed, in the pleasurable present moment as they appreciated the embodied, multisensory nature of looking out to sea. Results provide evidence that savouring the sea is experienced as an *active* process that reliably facilitates an interconnection between sea and self. Hence, findings are consistent with Bryant and Veroff’s (2007, p. 11) conception of savouring as “the capacity to attend to, appreciate, and enhance the positive experiences in one’s life”, and as an interaction between an individual and their environment. Analysis of diary data recorded during or immediately after sea gazing events also provides new insights that support Bryant and Veroff’s (2007) assertion that both external and self-focused mindfulness can coexist within the same savouring experience. Diary entries revealed that during a single sea gazing event individuals may shift multiple times between noticing and responding to external *and* internal stimuli; and that positive cognitive and emotional responses to both may be experienced as a consequence.

In this study, savouring the sea was compared to, but viewed as somewhat distinct, from the mechanisms of mindfulness and meditation. Similar to those practices, sea gazing was shown to involve mindful attention to thoughts and feelings in the present (Deikman, 1963; Kabat-Zinn, 1994). Indeed, the literature recognizes that mindfulness is an important part of savouring, as mindful awareness of the present is essential to be appreciative of it (Cheung & Ng, 2023). However, researchers have acknowledged that traditional mindfulness and meditation practices may not work for everyone, can be difficult to master and may promote negative outcomes (Delorme & Brandmeyer, 2018; Kaufmann et al., 2021; Schlosser et al., 2019; Van Dam et al., 2018). In contrast, for the women in this study, mindfully savouring the sea vista was experienced as effortless, infallible and solely focused on seeking out, and lingering upon, the pleasurable present. As such, savouring the sea seemed to unlock the ability to be “in the moment” for these women. Overall, these findings suggest that sea gazing may offer an effective and less effortful pathway to well-being for women in midlife who find it challenging to master traditional mindfulness and meditation techniques.

Within the proposed theory, engaging in proactive savouring is identified as one of the main pathways through which sea gazing positively affects well-being. Savouring the sea was shown to facilitate numerous positive and interrelated internal processes involved in emotional, cognitive and physiological release and relaxation, introspection, and self-transcendence. As a consequence of these processes, the women in this study experienced an improved sense of overall well-being, a reliable uplift in mood, and felt better able to cope with life stressors. These findings corroborate the claim that proactive savouring may be used as a regulatory mechanism to derive well-being benefits from positive events (Bryant, 2021; Cullen et al., 2024; Wilson & MacNamara, 2021); and support findings that savouring is associated with greater happiness, lower depression, and higher life satisfaction, particularly in older adults (Kurtz, 2018; Smith & Hollinger-Smith, 2015). The findings also provide evidence that savouring the sea may function as a coping response, supporting Samios et al. (2019) assertion that savouring may indirectly relate to better psychological adjustment through positive emotions following stressful life events. Researchers have found that interventions that facilitate savouring can increase psychological well-being and reduce negative emotions (Tri Kusuma & Yudiarso, 2022). However, few studies have focused on the potential benefits of *savouring the present* (rather than the past) interventions (Cullen et al., 2024), or naturalistic savouring experiences in everyday life (Bryant, 2021). Thus, there is an opportunity for future research to evaluate whether interventions that facilitate regularly savouring the sea have wider potential to promote well-being outcomes for women in midlife.

A second psychological mechanism identified as particularly significant within the proposed substantive theory is *connectedness to the sea*. Over time, participants in this study had developed a strong emotional, cognitive and, for some, spiritual connection to the sea which was maintained and enhanced through regularly sea gazing. Moreover, participants frequently speculated that connectedness to the sea might represent an innate predisposition rather than a learned behaviour. This finding is consistent with Wilson’s (1984) interpretation of the *biophilia hypothesis* that the human tendency to seek connections with the natural world may have a genetic basis. In addition, results highlighted that *re-connecting* to the sea while sea gazing not only facilitated feelings of greater connectedness to the sea itself, but also to the natural world as a whole, the wider universe, and to a personal interpretation of a “greater power.” In the present study, participants’ experience of sea connectedness was often explained as an unconditional *relationship*, in which the sea was perceived to perform multiple anthropomorphic roles. One interpretation of this finding is that the sea may help fulfil the basic psychological need for relatedness for women in midlife (Ryan & Deci, 2000). Indeed, the women in this study often described their bond with the sea as superior to many of their human relationships. Overall, findings suggest that individuals may develop a strong, enduring and multifaceted relationship with the sea, which can be reinforced by regularly gazing at the sea from the land.

In the present study, *connectedness to the sea* was identified as an important causal link between sea gazing and well-being. Re-connecting to the sea while sea gazing was perceived to promote hedonic (e.g., positive emotions and overall mood lift) and eudaimonic (e.g., a more balanced life view and greater resilience from a renewed sense of perspective) well-being outcomes. Overall, the findings are consistent with the concept of connectedness to nature as both an enduring trait, and a fluctuating state, that interact to promote well-being outcomes (Capaldi et al., 2014; Gál & Dömötör, 2023; Martin et al., 2020). Results also add to the growing body of correlational and experimental evidence that greater connectedness to nature is associated with benefits for emotional, mental, physiological and overall health (Capaldi et al., 2014; Gál & Dömötör, 2023; Jones & Wu, 2022, McEwan et al., 2019; Pritchard et al., 2020; Richardson & McEwan, 2018). To date, however, research into connectedness to natural environments has often not specified the type of nature being examined (e.g., Capizzi & Kempton, 2023) or has focused only on the well-being outcomes of connectedness to green spaces (e.g., Richardson & McEwan, 2018). However, the women in this study often described the sea as “unique in nature” and differentiated between their connectedness to the sea and their connection to other natural environments. Indeed, participants often claimed to feel more *inter*connected with the sea; and typically integrated the sea, and the act of sea gazing per se, within their emotional and cognitive representation of the self (Schultz, 2002). As such, findings build on suggestions in the qualitative literature that individuals can form a deeper emotional and spiritual attachment to the sea, which is not transferrable to other parts of nature (e.g., Diamond et al., 2024; Jarratt & Sharpley, 2017; Severin et al., 2022). Hence, future research should further explore the idea of a more multi-dimensional approach to nature connectedness, recognizing that people may differentially experience and benefit from connectedness to different parts of the natural world.

A third pathway through which sea gazing may promote well-being is via psychological and physiological restoration. In this study, sea gazing was claimed to reliably promote emotional, cognitive, and physiological release and relaxation, leaving participants feeling calmed, restored and re-charged. Regarding psychological restoration, sea gazing was shown to reliably promote an uplift in mood, enhance emotional regulation and lead to greater mental clarity. In terms of improving physiological markers of stress, looking out to sea was claimed to slow and deepen breathing, reduce stomach churning and chest tightness and lower perceived levels of adrenaline. Notably, these findings align with established medical models (Zakreski & Pruessner, 2019) indicating that participants may have experienced a shift in balance from the “fight or flight” response (sympathetic nervous system) to the “rest and digest” response (parasympathetic nervous system) while sea gazing. In addition, results are consistent with Ulrich et al. (1991) Stress Reduction Theory which states that natural landscapes containing views of water can reduce mental and physiological arousal. Findings also corroborate Kaplan and Kaplan’s Attention Restoration Theory (Kaplan & Kaplan, 1989), by providing insight into how savouring a cognitively undemanding natural stimulus—in this case the sea vista—can effortlessly and reliably clear the mind of “unwanted clutter” and so replenish the capacity for directed attention. Thus, findings from the present study add to the growing body of evidence that psychological and physiological restoration may be an important causal pathway linking blue spaces and well-being (for reviews see Georgiou et al., 2021; White et al., 2020).

Analysis of real-time and retrospective data within the present study highlighted that the main pathways linking sea gazing and well-being (i.e., proactive savouring, connectedness to the sea and restoration) seem to be experienced as interconnected rather than independent. For example, participants explained how proactively savouring the pleasurable present while sea gazing was perceived to facilitate mental, emotional and physiological relaxation and restoration, while simultaneously enhancing their feelings of connectedness to the sea. As such, findings support the view that specific natural settings may be linked with well-being via an intertwining of different mechanisms (Elliott et al., 2023; Hartig et al., 2014). However, previous blue space and well-being studies have typically focused on psychosocial pathways that originated in green space research, that is, restoration, physical activity and social connection (Georgiou et al., 2021; White et al., 2020). The current proposed theory, however, suggests that, of these three mechanisms, only restoration may be an important pathway linking sea gazing and well-being. Given the solitary nature of most sea gazing experiences, it is perhaps unsurprising that social connection may have little significance as a mechanism through which looking out to sea may impact well-being. In addition, whilst sea gazing was claimed to promote physical activity for some participants (e.g., by facilitating walking or running along the sea front), this pathway was not identified as an important link between sea gazing and well-being for all women in this study. Hence, the present findings may help explain why the literature is mixed regarding the mediating role of social connection and physical activity (Gascon et al., 2017; Georgiou et al., 2021; White et al., 2020). Overall, this study offers new evidence that connectedness to the sea, proactive savouring and psychological and physiological restoration may be the main pathways linking sea gazing from the land and well-being; and helps deepen our understanding of how these mechanisms are experienced by women in midlife.

### Limitations

The limitations of the present study should be acknowledged and used to inform future research. First, the proposed theoretical framework is a substantive theory (Corbin & Strauss, 2015) developed for a specific phenomenon (i.e., regular sea gazing) in a specific context (i.e., midlife women). Hence, some or all of the theory may not be relevant in other contexts. Future research could explore whether the substantive theory proposed has resonance in other fields. The present theory may provide a foundation for exploring similarities and differences with other phenomena and in other contexts, such as other proactive savouring experiences, different demographic groups and/or connectedness to other natural environments. Second, participants were selected for this study based on the criterion that they were already regular sea gazers. As such, the findings cannot be generalized to women in midlife who engage in this activity less often. Additionally, the results do not offer insight as to whether individuals within this age group who *do not* currently sea gaze would experience the same, or similar, well-being benefits were they to adopt this behaviour. Hence, future research in this field would benefit from exploring the beliefs and experiences of women who do, and do not, currently spend time sea gazing. Third, a strength of the present study was that retrospective interview data were supplemented with *in the moment* diary data to facilitate access to the thoughts, feelings, and experiences of participants while they were actively engaged in sea gazing. Nevertheless, participants only recorded their diary entries over a four-week period occurring sometime between February and June 2024. Given the potential impact of seasonal factors (such as weather, sea conditions and holidays) on the experience and outcomes of sea gazing, future research may find that collecting such data over a longer period of time yields additional insights.

## Conclusion and implications

In light of limited well-being research among middle-aged women, and growing evidence of the link between the sea and well-being, the present study aimed to explain the relationship between sea gazing from the land and well-being for coastal-dwelling women in midlife. The proposed substantive theory extends the recent work of Hurdman and Kampman (2024) and suggests that sea gazing is experienced as a reliable, self-reinforcing, cyclical process, that is habitually used as part of overall well-being management for this demographic. The findings of this study offer new insights into the important interconnected causal processes that may underpin the positive impact of this activity on the well-being of midlife women. Whilst previous researchers have also highlighted the role of the restoration pathway linking blue spaces with well-being, this study proposes two further causal mechanisms that are largely unexplored in the existing literature: proactive savouring and connectedness to the sea. In addition, the in-depth qualitative approach used in this study enhances our understanding of the *lived experience* of sea gazing from the land, and how this simple activity may be related to well-being. Whilst recognizing the limitations of this qualitative study, the findings indicate that one may not need to brave immersion in or on the water to reap the well-being benefits of the sea. Rather, results suggest that savouring the magic and majesty of the vista *from the land* may reliably restore, reset and enhance the well-being of women in midlife who regularly engage in this practice. Hence, this study helps to further our understanding of how different types of engagements with specific natural environments may be experienced and valued and may differentially impact well-being outcomes.

The results of this study may be of interest to policymakers and healthcare professionals. Nature-based interventions (NBI’s) have been shown to benefit clinical and non-clinical populations; however, their effectiveness has generally not been assessed with women in midlife. To date, most NBI’s have evaluated interventions taking place in green spaces (Haywood et al., 2021); or activities taking place in or on the water such as swimming, surfing and sailing (e.g., Hignett et al., 2018; Van Tulleken et al., 2018; Wilkie et al., 2022). However, such water-based activities have been argued to lack broad appeal, often require specific skills and experience, and are costly in time and money (e.g., Sotiriadou et al., 2020). In contrast, blue space interventions that involve sea gazing from the land may offer a low-cost and accessible way to obtain well-being benefits from the sea. Interventions that facilitate savouring have been found to increase psychological well-being (Tri Kusuma & Yudiarso, 2022), but few studies have focused on the potential benefits of *savouring the present* interventions or naturalistic savouring experiences in everyday life (Bryant, 2021; Cullen et al., 2024). Hence, NBI’s that facilitate savouring the sea are worthy of further exploration and may offer a pleasurable and effective way to promote well-being outcomes for women in coastal communities.

For women, midlife is often depicted as a time of inevitable psychological and physical change and decline (El Khoudary et al., 2019). Nevertheless, adopting positive health and well-being behaviours during this critical period has been shown to help optimize healthy functioning in older women (Avis et al., 2021). Whilst the present study proposes a theoretical explanation of how sea gazing from the land may positively impact well-being, it is recognized that more research is needed to determine if, how, and for whom, this activity has broader value as a well-being behaviour. Nevertheless, better understanding the well-being potential of spending time looking out to sea could have significant public health benefits for clinical and non-clinical populations of women in midlife.

## Supplementary Material

Clean copy ZQHW-S-2025-0059.R2.docx

## Data Availability

Anonymised and de-identified data associated with this paper (i.e., transcripts of interviews and diary entries) is available on request by emailing the corresponding author at s.l.hurdman@sussex.ac.uk
